# ClaID: a Rapid Method of Clade-Level Identification of the Multidrug Resistant Human Fungal Pathogen Candida auris

**DOI:** 10.1128/spectrum.00634-22

**Published:** 2022-03-28

**Authors:** Aswathy Narayanan, Pavitra Selvakumar, Rahul Siddharthan, Kaustuv Sanyal

**Affiliations:** a Molecular Mycology Laboratory, Molecular Biology and Genetics Unit, Jawaharlal Nehru Centre for Advanced Scientific Research, Bangalore, Karnataka, India; b Computational Biology, The Institute of Mathematical Sciences, Chennai, Tamil Nadu, India; c Homi Bhabha National Institute, Mumbai, Maharashtra, India; d Osaka University, Suita, Osaka, Japan; University of Minnesota Medical School

**Keywords:** colony PCR, clade-specific sequences, sequence junctions, diagnostic method, antifungal resistance, evolution, fungi

## Abstract

Candida auris, the multidrug-resistant human fungal pathogen, emerged as four major distinct geographical clades (clade 1–clade 4) in the past decade. Though isolates of the same species, C. auris clinical strains exhibit clade-specific properties associated with virulence and drug resistance. In this study, we report the identification of unique DNA sequence junctions by mapping clade-specific regions through comparative analysis of whole-genome sequences of strains belonging to different clades. These unique DNA sequence stretches are used to identify C. auris isolates at the clade level in subsequent *in silico* and experimental analyses. We develop a colony PCR-based *cla*de-*id*entification system (ClaID), which is rapid and specific. In summary, we demonstrate a proof-of-concept for using unique DNA sequence junctions conserved in a clade-specific manner for the rapid identification of each of the four major clades of C. auris.

**IMPORTANCE** C. auris was first isolated in Japan in 2009 as an antifungal drug-susceptible pathogen causing localized infections. Within a decade, it simultaneously evolved in different parts of the world as distinct clades exhibiting resistance to antifungal drugs at varying levels. Recent studies hinted the mixing of isolates belonging to different geographical clades in a single location, suggesting that the area of isolation alone may not indicate the clade status of an isolate. In this study, we compared the genomes of representative strains of the four major clades to identify clade-specific sequences, which were then used to design clade-specific primers. We propose the utilization of whole genome sequence data to extract clade-specific sequences for clade-typing. The colony PCR-based method employed can rapidly distinguish between the four major clades of C. auris, with scope for expanding the panel by adding more primer pairs.

## INTRODUCTION

Fungal infections pose a serious threat in the health care sector due to high mortality rates and limited antifungals available for treatment, compounded by the emergence of new species accelerated by environmental changes, including global warming (1). Candida auris, a recent addition to the list of opportunistic human fungal pathogens, was first isolated from an infected ear of a patient in 2009 in Japan (2). In the past decade, it transformed into a global public health concern, emerging as disparate geographical clades (3). C. auris causes life-threatening bloodstream infections, which result in mortality rates ranging from 30% to 60% (4). In the last 2 years, a high incidence of C. auris co-infections was reported in COVID-19 patients (5).

Factors that caused C. auris to become a superbug include its ability to cause nosocomial outbreaks, resistance to the existing antifungal classes, and lack of a fast, sensitive, and reliable diagnostic tool to identify it accurately at the species level (https://www.cdc.gov/fungal/candida-auris/c-auris-drug-resistant.html). C. auris was initially misidentified as Candida haemulonii, a closely related species, by the existing yeast identification systems (6). Diagnostics for detecting C. auris have evolved rapidly in the past decade. At the species level, it can be distinguished from its close relatives by sequencing the internal transcribed spacer (ITS) regions or specific ORF sequences like GPI-encoding genes or pyruvate: ferredoxin oxidoreductase domain (7–9). Culture-dependent methods of identification include biochemical systems and culture media (10). Diverse techniques including real-time PCR, loop-mediated isothermal amplification (LAMP), T2 magnetic resonance (T2MR), and oligonucleotide-capped nanoporous anodic alumina (NAA) biosensors have been employed for the detection of C. auris isolates (7, 9, 11, 12). In addition to species identification, allele-specific molecular beacons were also designed for allele-specific PCR to detect mutations in the genes *ERG11* and *FKS1*, which are associated with azole and echinocandin resistance, respectively (13).

While all these methods can be employed to identify species, they are unable to distinguish isolates of different geographical clades. Years after its first isolation in 2009, whole-genome sequencing of multiple C. auris clinical isolates revealed its simultaneous emergence in various geographical regions. C. auris isolates were then categorized as geographical clades- clade 1 (South Asian), clade 2 (East Asian), clade 3 (South African), and clade 4 (South American) (2, 3). Strains belonging to different clades also exhibit distinct properties. Clades 1 and 4 have MTL**a**, while clades 2 and 3 possess MTL*α* at the mating-type locus (14). Isolates belonging to clade 3 form large aggregates compared to isolates from the other clades, while pseudohyphae formation is pronounced in South Asian isolates (15). When tested in a neutropenic murine bloodstream infection model, mortality rates were the highest for clade 4 isolates, followed by clades 1, 3, and 2 (16). The higher mortality caused by South American isolates was also observed in *Drosophila* (17). These observations are suggestive of variations in virulence-associated features between the clades. Except for clade 2 isolates that are generally drug-susceptible, C. auris clinical isolates exhibit resistance to multiple drug classes including azoles and polyenes (18, 19). The clades exhibit marked differences in their response to various antifungals (15, 20), with drug resistance-associated single nucleotide polymorphisms (SNPs) showing a clade-based bias (14). These observations emphasize the need for identification at the clade level.

Initially, the strains isolated in the same geographical zone exhibited clustering, on whole genome sequence analysis (18). However, subsequent reports suggest dissemination of strains - isolates obtained in Russia clustered with South Asian clade strains and isolates from the UK were of multiple geographical origins (21, 22). Strains belonging to clades 1, 2 and 4 were obtained in Singapore, while clades 2, 3 and 4 strains were isolated in South Africa (23, 24), suggesting mixing of clades in different geographical zones. These reports also hint that the area of isolation will soon cease to be a reliable basis of clade-typing.

Clade-typing largely relies on whole genome sequencing and multilocus sequence typing (3, 18, 25), which can be time-consuming. Previously reported, clade-specific identification using short tandem repeats (STR) involves a sequencing step (26). Allele-specific PCR (AS-PCR) based on conserved mutations in the internal transcribed spacer sequences reported recently (27), relies on multiple reactions to differentiate the clades properly.

In this study, we identify conserved clade-specific sequences for rapid *cla*de-*id*entification (ClaID) and utilized them for single amplification reactions corresponding to each clade. This results in a rapid and specific method for identification of C. auris at the clade-level.

## RESULTS

### The genome-level comparison identifies clade-specific sequences.

**FIG 1:**
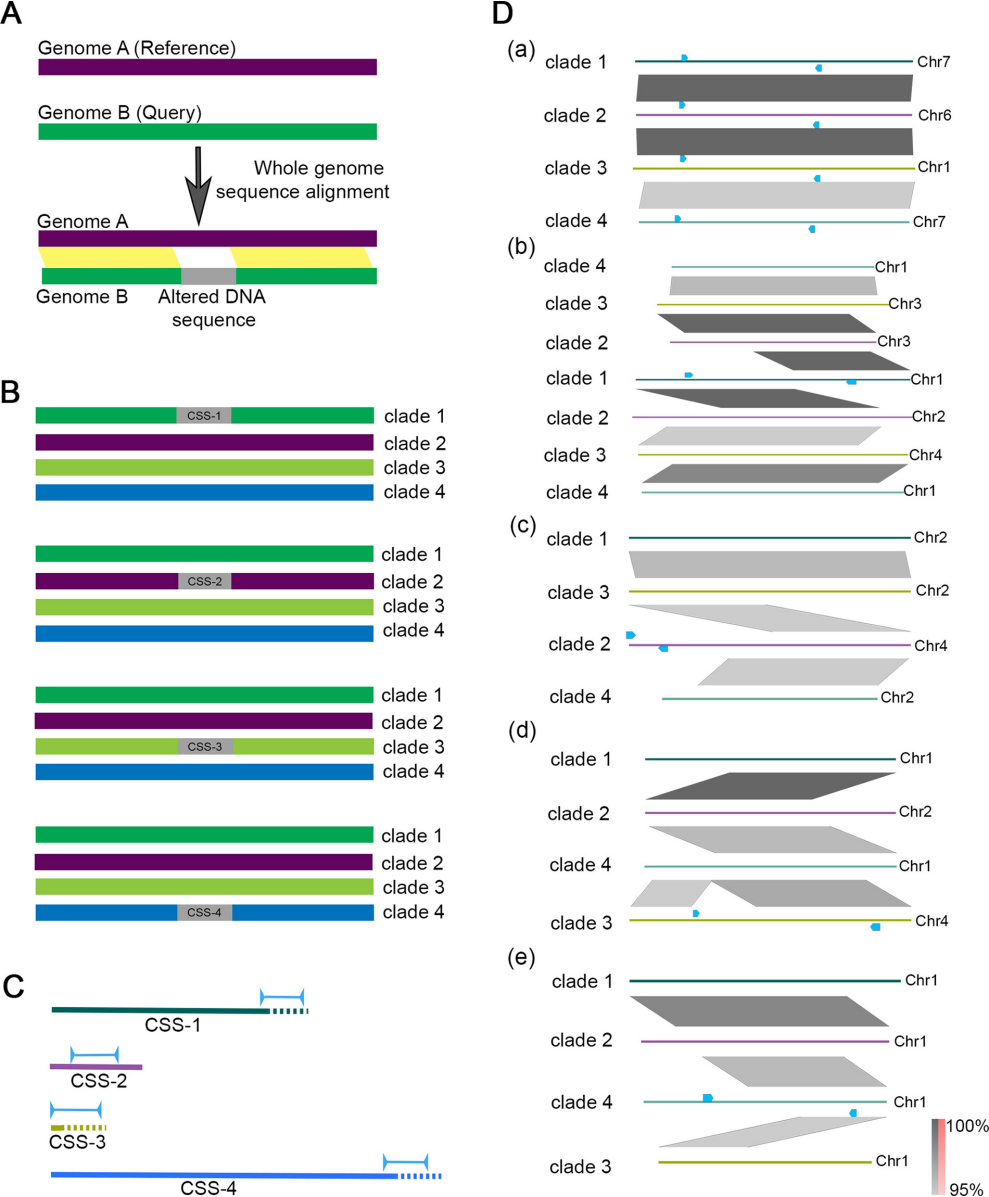
Clade-specific DNA sequences were identified *in silico* for clade-typing in C. auris. A. Pictorial representation of identifying altered DNA sequences through whole genome alignment using the tool, NUCmer. The sequence stretches aligned between the query and the reference genomes are shown in yellow. The unaligned (altered) sequence is considered as the unique clade-specific sequence (CSS) (shown in gray). B. Schematic depicting the presence of clade-specific sequences in each of the four clades (CSS-1 to CSS-4) of C. auris, identified using all possible combinations of pairwise whole genome alignments. The genomes are shown as solid lines, and the clade-specific sequences are shown in gray. C. Schematic showing the locations and orientations of the primers and amplicon sequences (blue) in the unique sequences identified (solid lines) with respect to the flanking sequences (dashed lines) in each clade. Coordinates of the unique sequences and amplicon sequences are given in Table 1. D. Presence of AUS in each of the four clades is shown in (a). Clade-specificity of (b) CSS-1 (c) CSS-2 (d) CSS-3, and (e) CSS-4 is also shown. The neighborhood of CSS-1 through CSS-4 across the four clades are shown. Chromosomal regions of different clades are shown in distinct colors. The primer positions and orientations are indicated by arrows in blue. Sequences showing homology are shown in gray, and inversions (if present) are shown in red. The extent of homology is shown on the color scale. The chromosomal coordinates used for generating the figure are given in Supplementary Information.

A universal primer pair was designed to amplify sequences from all the four clades, denoted as auris universal sequence (AUS) (Table 1). Once the fungal pathogen is identified as C. auris, identification at the clade level can be done using clade-specific sequences identified *in silico*. Briefly, genome assemblies of representative strains of each of the four clades were analyzed using NUCmer, a tool for whole genome sequence alignment (see Materials and Methods, Fig. 1A). All possible combinations of pairwise alignments of four genome assemblies, each representing one of the four major geographical clades, were performed. The sequence hits obtained under several categories like inversions, breaks, and gaps were analyzed. DNA sequence alterations specific to a clade but absent in the other three clades were shortlisted (Fig. 1B). The DNA sequences that showed specificity in initial homology searches were chosen for designing clade-specific primers. The amplicon sequences were selected from the unique sequences or sequence junctions (Fig. 1C) and were individually analyzed using homology searches to confirm specificity, following their identification (Table 1).

**TABLE 1:** Oligonucleotide primers used to amplify clade-specific DNA sequences

Sequence	Purpose	CSS chromosomal coordinates^b^ (length in bp)	PCR amplicon coordinates (length in bp)	Primer sequences^a^
AUS	Species-identification		Clade 1: CP060345.1 289128-289573 (446) Clade 2: CP043536.1 525555-526000 (446) Clade 3: CP060367.1 3254166-3254592 (446) Clade 4: CP043442.1 278974-279419 (446)	FP-AGAGTCGAGTGAGTCAAAAC RP-CTCAACTCGGAATTTTTCATC
CSS-1	Clade-identification	CP060339.1 901357-905181 (3825)	CP060339.1 904959-905458 (500)	FP-TTATTTGGTCTTCAATCATTGATTCCTTGC RP-TACGTGTAGTGAGTAGGAATTGAGG
CSS-2	Clade-identification	CP043534.1 13696-15335 (1640)	CP043534.1 14050-14600 (551)	FP-AGCTACACAAAATGGTTTTTTCAGAT RP-CACATCATATGCCAAAGTAGTAGAGT
CSS-3	Clade-identification	CP060370.1 287700-287929 (229)	CP060370.1 287703-288300 (598)	FP-CGATGAGAAACCCCCATCCAA RP-TTTTCATTTCTATCAGTCAATACAATACGACC
CSS-4	Clade-identification	CP043442.1 3690760-3697046 (6287)	CP043442.1 3696899-3697400 (502)	FP-GGGGGTTTTACTATATAAATTTGTATAGCTT RP-CTATGTAGGTCGGGATTTTCATCC

a FP: forward primer, RP: reverse primer.

b Coordinates correspond to the respective assemblies mentioned in Materials and Methods.

The category in which the DNA sequences were identified gave a preliminary idea about the underlying structural change. Unaligned sequences are categorized by NUCmer into different groups: GAP (a gap between two mutually consistent alignments), DUP (inserted duplication), JMP (shifting of alignment allowing insertion of a unique sequence), INV (rearrangement with inversion), and SEQ (rearrangement with another sequence). The clade 1 sequence block chosen for designing primers was identified in the analysis as an inversion event where the alignment orientation is disrupted. Clade 2-specific sequence was acquired as an output of the function SEQ, which reports a translocation event that requires jumping to a new query sequence to continue aligning to the reference. Clade 3 sequence was identified by the GAP function, representing a gap between two mutually consistent ordered and oriented alignments. The specific sequence for clade 4 was reported by the function JMP, which identifies sequence insertion events where the consistent ordering of alignments is disrupted. These events were also visualized using dot plots (Fig. S1 in the online supplemental material).

On closer examination of the DNA sequences, it was clear that none of the sequences chosen were present as contiguous stretches except for the clade in which they were identified (Fig. 1D). Each clade-specific sequence represents a breakpoint in an otherwise consistent region of alignment. The breakpoints are categorized depending on the alignment that precedes or succeeds them. CSS-1 is located at the breakpoint that borders an inversion. In clade 4, CSS-1 is present on the same chromosome, Chr1, as two blocks with coordinates 887976–888314 and 2150717-2150542. As the first block is separated from the second block by 1.3 Mb, they will not be amplified by the designed primers. CSS-1 amplicon is split into two blocks present on two different chromosomes in clades 2 (Chr2 and Chr3) and clade 3 (Chr3 and Chr4). CSS-2 and CSS-3 amplicon sequences are present exclusively in clades 2 and 3, respectively. CSS-4 represents the shifting of an aligned sequence, showing the insertion of a unique sequence. Only a part of the CSS-4 amplicon sequence was conserved in the other three clades (supplementary information).

### Design of ClaID.

**FIG 2:**
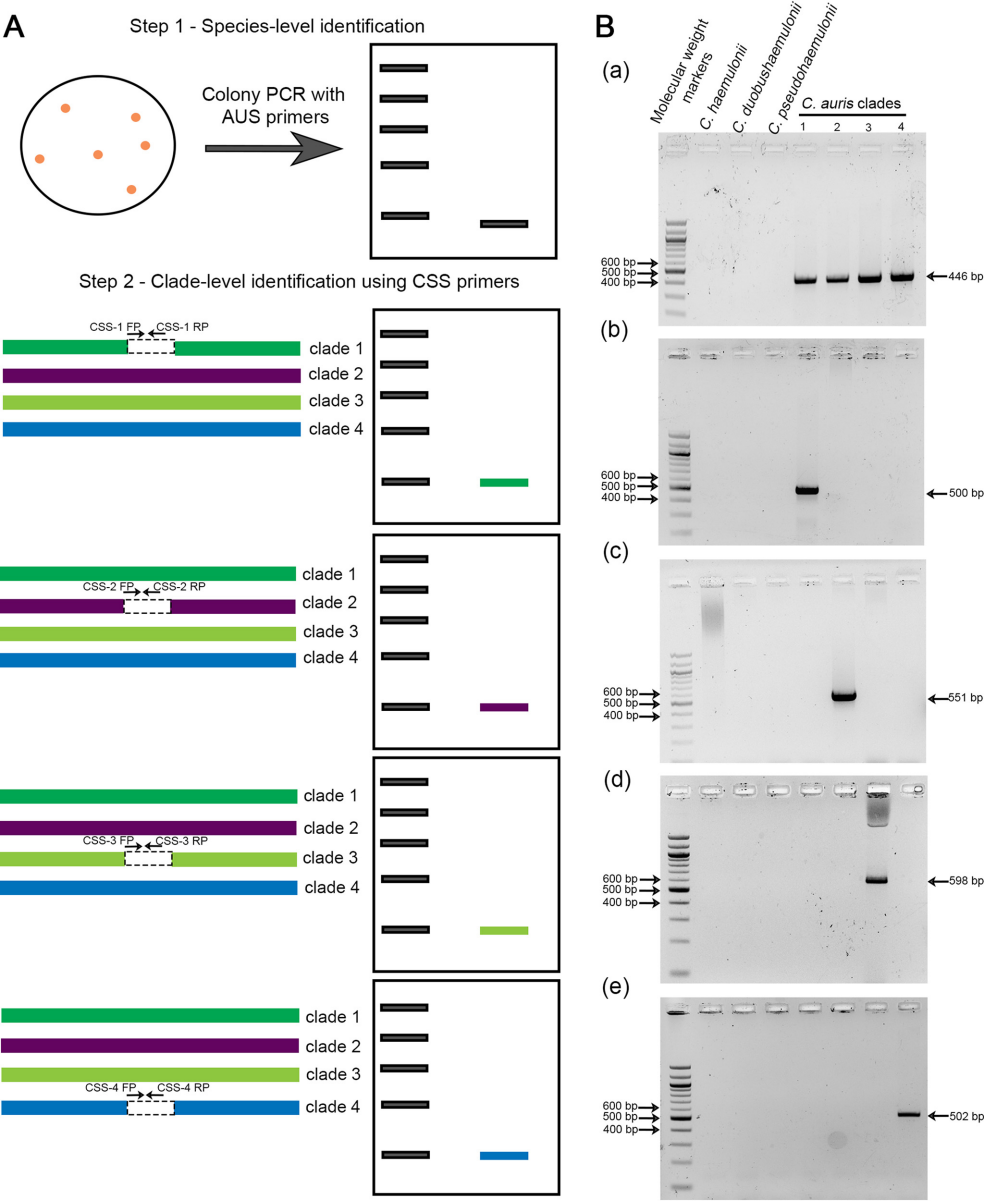
Clade-specific sequences (CSS) act as targets for unique clade-specific PCR amplifications. A. Schematic showing the design of ClaID. Step 1, Species-level identification using the auris universal sequence (AUS) primers in colony PCR. Step 2, Clade-level identification using CSS primers. The genomes of various clades are shown as indicated, and the clade-specific sequences are shown in gray. The primer positions and directions are marked by black arrowheads. B. PCR amplifications obtained using (a) AUS primers that amplify from all the clades (b) CSS-1, specific to clade 1 (c) CSS-2, specific to clade 2, (d) CSS-3, specific to clade 3, and (e) CSS-4, specific to clade 4. Molecular markers ranging from 100 bp to 1.5 kb is loaded in lane 1, PCR products from reactions using different genomic DNA samples are loaded in lanes 2–8 as indicated.

ClaID, the PCR detection system, has five pairs of primers: (i) a universal primer pair designed from a DNA sequence conserved across the clades to confirm the species as C. auris. (ii) Four pairs of clade-specific primers designed from DNA sequences or sequence junctions that are clade-specific. The amplicon sizes were designed to be around 500 bp to increase the amenability to colony PCR, thereby bypassing genomic DNA extraction and facilitating rapid detection (Fig. 2A). AUS was tested against genome assemblies and isolates of closely related species *C. haemulonii*, *C. duobushaemulonii*, and *C. pseudohaemulonii*. The preliminary tests of clade-specific sequences were done against four representative isolates of each clade (Fig. 2B).

### ClaID is fast, reproducible, and specific.

To confirm the reproducibility of the PCR amplification, 10 colonies of the tested strains were independently amplified, and specific PCR amplification for the corresponding clade was obtained (Fig. S2 in the supplemental material). No cross amplifications were observed, suggesting that the detection system is accurate and robust. Sanger sequencing confirmed the identity of the DNA sequences of all the amplicons. The scope of using the primers across strains was confirmed by testing the sequences against the publicly available genome assemblies that include strains from all the clades using BLAST. A query coverage of 100% was considered as positive hits, as this includes both the primer sequences and the amplicon sequence flanked by them. One of the amplicon sequences, excluding the primer sequences, showed sequence variation; hence, the percentage identity selection was relaxed, given the primer sequences were conserved (Table 2). The reverse analysis also confirmed the specificity of the sequences- publicly available assemblies of strains with known clade status were probed with all the primer sequences: AUS and CSS. Except for BJCA001, which was reported as a clade 1 isolate (28), all other assemblies tested showed specificity, conforming to the known clade status (Table 3). All the assemblies analyzed had predicted amplicon for AUS.

**TABLE 2:** Homology search for the clade-specific sequences

Sequence	Strains	Clade	GenBank assembly
CSS-1	CA7LBN	1	GCA_019332045.1
	CA2LBN	1	GCA_019039775.1
	CA5LBN	1	GCA_019039755.1
	CA4LBN	1	GCA_019039735.1
	CA6LBN	1	GCA_019039715.1
	CA9LBN	1	GCA_019039695.1
	CA12LBN	1	GCA_019039675.1
	CA16LBN	1	GCA_019039655.1
	CA8LBN	1	GCA_019039635.1
	CA10LBN	1	GCA_019039615.1
	CA1LBN	1	GCA_019039595.1
	CA11LBN	1	GCA_019039575.1
	CA18LBN	1	GCA_019039555.1
	CA17LBN	1	GCA_019039535.1
	CA14LBN	1	GCA_019039515.1
	CA23LBN	1	GCA_019039495.1
	CA13LBN	1	GCA_019039475.1
	CA24LBN	1	GCA_019039455.1
	CA22LBN	1	GCA_019039435.1
	CA25LBN	1	GCA_019039415.1
	CA15LBN	1	GCA_019039395.1
	CA19LBN	1	GCA_019039375.1
	CA26LBN	1	GCA_019039355.1
	CA27LBN	1	GCA_019039335.1
	CA28LBN	1	GCA_019039315.1
	CA20LBN	1	GCA_019039295.1
	CA21LBN	1	GCA_019039275.1
	CA29LBN	1	GCA_019039235.1
	B13916	1	GCA_016772235.1
	B11205	1	GCA_016772135.1
	CA-AM1	1	GCA_014673535.1
CSS-2	B11220	2	GCA_003013715.2
	B12043	2	GCA_016495645.1
	B11809	2	GCA_016495685.1
	B13463	2	GCA_016495665.1
CSS-3	B12037	3	GCA_016772215.1
	B12631	3	GCA_016772195.1
	B17721	3	GCA_016772175.1
CSS-4	B11245	4	GCA_008275145.1
	B12342^*a*^	4	GCA_016772155.1

a Sequence variations within the amplicon sequence were detected (query coverage: 100%, percentage identity: 96), though the primer sequences were conserved.

**TABLE 3:** Assembly-based confirmation of clade-specificity^*a*^

Strain	AUS	CSS-1	CSS-2	CSS-3	CSS-4	Inferred clade status	Known clade status
BJCA002	Y	N	N	Y	N	clade 3	clade 3
CAU924	Y	Y	N	N	N	clade 1	clade 1
LOM	Y	N	N	Y	N	clade 3	clade 3
BJCA001	Y	N	N	N	N	Not determined	clade 1
A1	Y	N	N	Y	N	clade 3	clade 3
JCM1448	Y	N	Y	N	N	clade 2	clade 2
B11221	Y	N	N	Y	N	clade 3	clade 3

a Y denotes presence of predicted PCR amplicon; N denotes its absence.

A collection of clinical isolates was tested using all sets of primers, in addition to all the strains screened *in silico* using homology searches to assess the applicability of the ClaID detection system across strains (Table 4). This collection consisted of six clade 1 isolates from India, one clade 2 Japanese isolate (CBS1091131T), one clade 3 isolate (598A), and one clade 4 isolate (LMDM1219). Our analyses confirmed that all six clinical samples isolated in India belong to clade 1, the South Asian cluster. The strains 598A and LMDM1219 were confirmed to be a clade 3 and clade 4 isolate, respectively (Fig. S2). Combining the *in silico* and experimental analyses, 4/4 clade 2 isolates, 9/9 clade 3 isolates, and 4/4 clade 4 isolates tested could be detected accurately using ClaID, indicating a 100% specificity. For clade 1, 38 out of 39 isolates tested could be identified correctly, suggesting a 97.4% specificity.

**TABLE 4:** Strains used in the study

Species	Strains	Reference
C. auris clade 1	Cau46R, 470149, 470147, 470154, 470100, 470155, 470097, 470150, 470055	(29), NCCPF*^a^* collection
C. auris clade 2	CBS1091131T	(29)
C. auris clade 3	598A, AR-0383	(29) for AR-0383
C. auris clade 4	LMDM1219, AR-0385	(29) for AR-0385
C. haemuloni i	NCCPF470162	(29)
C. duobushaemulonii	NCCPF470164	(29)
C. pseudohaemulonii	NCCPF470163	(29)

a National Culture Collection of Pathogenic Fungi.

## DISCUSSION

C. auris evolved as disparate geographical clades during the last decade. In this study, we have identified clade-specific DNA sequence junctions based on the pairwise alignment of whole-genome sequences of representative assemblies belonging to the four major geographical clades. The proposed colony PCR-based strategy, ClaID, relies on the amplification of approximately 500 bp of DNA sequences designed for clade-typing and was tested in clinical isolates to obtain clade-specific amplifications, with very high specificity.

Chromosomal rearrangements can shape the genome of an emerging species, delineating it from closely related species. In addition, such genome alterations contribute toward generating diversity within a species by introducing interclade karyotype variations, as shown previously in different geographical clades of C. auris (29). We reasoned that some of the junctions resulting from rearrangements might be conserved in a clade-specific manner. We tested this hypothesis using multiple assemblies of C. auris strains belonging to the four major clades, available in the public domain.

We identified sequences and sequence junctions unique to each clade by pairwise genome alignments. Both *in silico* and experimental analyses across assemblies and strains confirmed the specificity of the identified clade-specific sequences. Though the recent clade, clade 5 from Iran (30), was not included in the current panel, we did not obtain any hits from clade 5 in the homology searches, confirming that the selected CSS primer sequences are unlikely to yield any amplification from clade 5. The clade 5 assembly (GCA_016809505.1) gave a putative amplicon when queried with AUS, confirming the potential to use AUS across different clades.

The existing techniques that can distinguish between C. auris clades either require DNA sequencing or multiple PCR amplification reactions. The rapid spread and mixing of strains belonging to different clades in a specific geographical location call for rapid detection techniques that are reliable, fast and does not involve a DNA sequencing step. A detection method that provides a readout with minimum number of reactions will enable faster and efficient clade-typing. The amplification reactions in ClaID described in this study are fast, specific, reliable, and reproducible. A pan-clade AUS primer pair of C. auris confirms the identity of the species and acts as a filter before proceeding to clade-specific amplification reactions. The detection strategy requires a fungal culture, limiting its use in clinical samples. To improve the turnaround time, the amplicon sizes are designed to be around 500 bp, thus reducing the extension time required. Additionally, all the primer pairs were tested multiple times for amenability to colony PCR to bypass the step of genomic DNA isolation. The identification strategy does not depend on DNA sequencing; the presence of the amplicon indicates the clade identity of the strain. We did not obtain any false positives confirming the accuracy of the detection system.

Detection of mixing of geographical clades at different locations will help in tracking the spread of C. auris. ClaID can thus serve as a useful epidemiological tool by detecting the presence of strains belonging to different clades in a single location. Though restricted primarily to intensive care units and other clinical settings, C. auris was recently isolated from the virgin habitats of The Andaman and Nicobar Islands, indicative of a broader distribution that possibly includes environmental niches (31). Although no evidence for meiosis in C. auris could be detected yet, a complete sexual cycle is elucidated in Candida lusitaniae, a closely related species (32). Isolation of C. auris strains belonging to different clades of opposite mating types in the same geographical location raises the possibility of sexual interaction among the clades, genetic recombination, and ultimately generation of new strains (33). Therefore, we cannot rule out the emergence of new strains or new clades that cannot be typed using the existing system considering the rapid evolution of C. auris as a pathogen. This study employed a single primer pair for each clade to prove the concept of how signature chromosomal rearrangements and resulting junctions can be utilized for typing geographical clades if supported by analysis of a large data set. Multiple primer pairs can be designed similarly to expand the panel accommodating more clade-specific sequences to improve the detection range, taking the recombination potential into consideration. Several pathogens like C. albicans and C. glabrata exist as geographical clades, with clade-specific phenotypic features (34–36). This strategy can also be extended to other species of clinical interest.

## MATERIALS AND METHODS

### Strains used in the study.

Strains and clinical isolates used in the study are listed in Table 4. The strains were grown in YPD medium (1% yeast extract, 2% peptone, and 2% dextrose) at 30°C.

### Genome-level sequence comparison.

A pairwise alignment of representative genomes of the four clades was done to determine clade-specific breakpoints among the four Candida auris clades. The genome assemblies of strains belonging to clade 1 (https://www.ncbi.nlm.nih.gov/assembly/GCA_016772135.1), clade 2 (https://www.ncbi.nlm.nih.gov/assembly/GCA_003013715.2), clade 3 (https://www.ncbi.nlm.nih.gov/assembly/GCA_016772215.1), and clade 4 (https://www.ncbi.nlm.nih.gov/assembly/GCA_008275145.1) as on 11 February 2022 were used as representatives for the analysis.

MUMmer’s NUCmer (NUCleotide MUMmerprogram) (v.3.9.4 alpha) (37) was used to align two genomes belonging to different clades. NUCmer first uses MUMmer to find all the unique matches (with respect to the reference sequence) of a given length between the two input sequences. Individual matches that fall within a gap and match threshold are grouped in a clustering step using the mgaps algorithm. This step results in larger sets of consistently ordered (non-exact) matches. Finally, the clusters are aligned to increase the overall coverage using a modified version of the Smith-Waterman algorithm. The output of NUCmer is a delta file that catalogs the coordinates of each alignment and notes the distance between insertions and deletions contained in these alignments. show-diff, which is also a part of the MUMmer package, generates a list of structural differences for each sequence in the reference and query, sorted by position, using the output from NUCmer. For a reference sequence R and its matching query sequence Q, differences are categorized as GAP (a gap between two mutually consistent alignments), DUP (inserted duplication), BRK (other inserted sequence), JMP (shifting of alignment allowing insertion of a unique sequence), INV (rearrangement with inversion), and SEQ (rearrangement with another sequence). The nonaligned regions with respect to the reference genome are reported.

All combinations of pairwise alignments were performed to identify the clade-specific sequences (CSS) by taking one of the assemblies as the reference and the other as the query. NUCmer was run with the reference and query genome files (in multi-FASTA format) with the default parameter values. The output delta format file was then used as input for show-diff analysis. bedtools getfasta (v2.29.2) (38) was used to retrieve the sequences corresponding to the output feature coordinates of show-diff (INV, SEQ, GAP, JMP, BRK, DUP) from the respective reference genome. BLASTn (v2.10.1) (39) program was run by taking the retrieved sequences as query sequences to look for alignments in the four representative genomes to know if any particular sequence is unique and specific to only the clade representative genomes.

The results were pooled into a table wherein the query alignments in the four genomes were indicated by the number of hits returned by BLASTn. Out of multiple putative sequences, one set of specific sequences for each clade- four sequences, each belonging to a different category (INV, SEQ, GAP, and JMP)- was chosen from the table. Multiple primers were designed for amplifying contiguous sequences of the length of 500 bp approximately. A preliminary homology search using different amplicon sequences revealed clade-specificity of a few amplicons which were chosen for primer design. The selected sequences were analyzed across the four clades for each assembly and were visualized using EasyFig (40). NUCmer results were plotted using the delta file to better visualize the structural differences of breakpoints. The delta file contains information on the distances of subsequent indels in each cluster of matches that can be used to extract aligned regions. The alignment regions were plotted using ggplot2 in R.

### *In silico* confirmation of clade-specificity.

Each CSS identified was used as a query against the nucleotide collection and for species-specific homology search using BLAST. Strains of known clade status that did not appear in the BLAST output were analyzed separately for the presence of the amplicon sequence. Primers were designed from the selected sequences and tested against representative isolates of the four clades and the closely related C. haemulonii species complex.

### Genomic DNA isolation and PCR.

Genomic DNA was extracted from yeast cells grown in YPD using the glass bead method. Briefly, approximately 2 O.D cells were collected, and an equal volume of sterile glass beads was added to the pellet, along with 500 μL of extraction buffer (2% Triton-X, 1% SDS, 100 mM NaCl, 100 mM Tris pH 8.0, and 1 mM EDTA). The sample was vortexed for 3 min and was incubated at 65°C for 10 min. Subsequently, 0.6 mM potassium acetate (pH 5.2) and 0.5 mM sodium chloride were added, and the sample was incubated in ice for 10 min. The samples were then centrifuged at 16000g for 10 min, and the supernatant was collected in a fresh tube. Genomic DNA was precipitated using 2.5 times the volume of absolute alcohol and one-tenth volume of 3M sodium acetate, air-dried, and dissolved in water. Multiple washes with 70% ethanol were followed by RNase treatment and phenol-chloroform-isoamyl alcohol extraction.

A total reaction volume of 20 μL consisted of approximately 40 ng of the template DNA, 2 μL of the 10 X *Taq* Buffer supplemented with 25 mM MgCl_2_ (Genei), 0.6 U of *Taq* DNA polymerase (Genei), 2 μL of 2.5 mM dNTPs (Thermo Scientific), and 10 pm of the forward and reverse primer. The initial denaturation step was performed at 95°C for 3 min, followed by 30 cycles of denaturation at 95°C for 30 s, annealing at the respective T_m_ for 30 s, and extension at 72°C for 45 s. A final extension step was done at 72°C for 10 min. The amplicons were analyzed on a 2% agarose gel (Seakem LE agarose, Lonza) and imaged under UV-light using Chemidoc Imaging System (Bio-Rad).

### Colony PCR.

Fresh colonies growing on YPD (1% Yeast-extract, 2% peptone, and 2% dextrose) were picked and suspended in 30 μL of 0.2% SDS. After brief vortexing, the cells were incubated at 90°C, for 5 min in a dry bath. This step was followed by vigorous vortexing. The cell debris was removed as a pellet after centrifugation at 16,000 g for 3 min. One microliter of the supernatant was used as the template for the amplification reactions.

A total reaction volume of 50 μL consisted of 1 μL of the template DNA, 5 μL of the 10 X *Taq* Buffer supplemented with 25 mM MgCl_2_ (Genei), 1.5 U of *Taq* DNA polymerase (Genei), 4 μL of 2.5 mM dNTPs (Thermo Scientific), 2 μL of 25% Triton-X (Sigma), and 15 pm of the forward and reverse primer. The initial denaturation step was performed at 95°C for 3 min, followed by 35 cycles of denaturation at 95°C for 30 s, annealing at the respective T_m_ for 30 s, and extension at 72°C for 45 s. A final extension step was done at 72°C for 10 min. The amplicons were analyzed on a 2% agarose gel (Seakem LE agarose, Lonza) and imaged under UV-light using Chemidoc Imaging System (Bio-Rad).
