# Supplementary material for: ClaID: a Rapid Method of Clade-Level Identification of the Multidrug Resistant Human Fungal Pathogen Candida auris
Source: Microbiol Spectr. 2022 Mar 28;10(2):e00634-22. doi: 10.1128/spectrum.00634-22 (PMC9045239; doi:10.1128/spectrum.00634-22)
Supplement: SUPPLEMENTAL FILE 1 — Supplemental material. Download SPECTRUM00634-22_Supp_1_seq3.pdf, PDF file, 0.6 MB [file spectrum00634-22_supp_1_seq3.pdf]

Supplemental Material

ClaID: A rapid method of clade-level identification of the multidrug resistant human fungal pathogen *Candida auris*

Aswathy Narayanan[1, #], Pavitra Selvakumar[2,3], Rahul Siddharthan[2,3], Kaustuv Sanyal[1,4, #]

[1] Molecular Mycology Laboratory, Molecular Biology and Genetics Unit, Jawaharlal Nehru Centre for Advanced Scientific Research, Bangalore, India; [2]Computational Biology, The Institute of Mathematical Sciences, Chennai, India; [3] Homi Bhabha National Institute, Mumbai, India; [4] Osaka University, Suita, Osaka, Japan

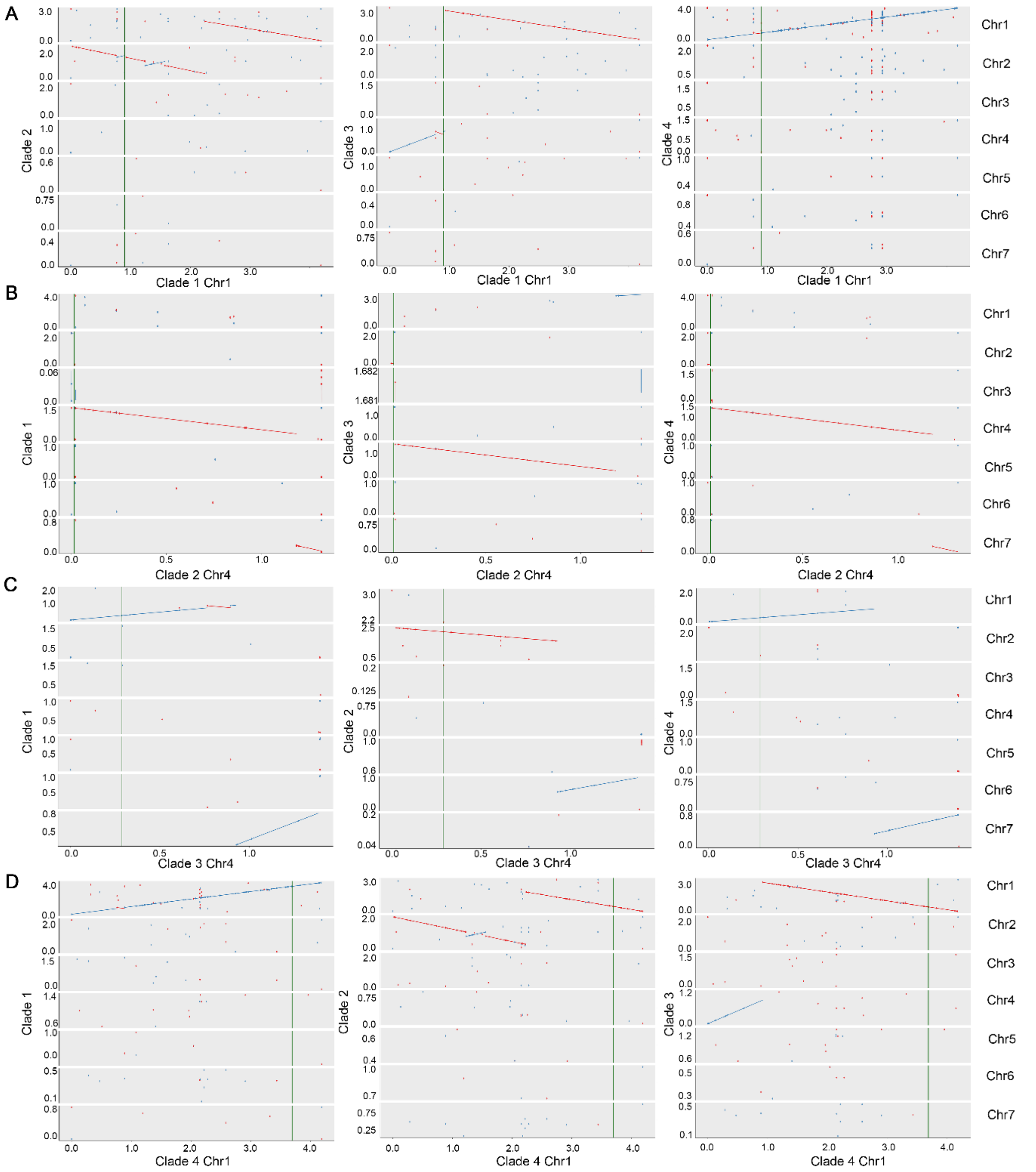

FIG S1 The figure shows the alignments in the query clade contigs from NUCmer's output (delta file) corresponding to the reference contig containing the clade-specific sequence. The x-axis represents the contig from the clade taken as reference, and the y-axis shows the clade taken as the query. Alignments in the same orientation in reference and query are shown in blue, and those with opposite orientation (inversions) are shown in red. The region shown by the green line corresponds to the unique clade-specific sequence in the reference

clade. The chromosome coordinates are marked on both the axes, in Mb. A. Chromosome 1 in clade 1 B. Chromosome 4 in clade 2 C. Chromosome 4 in clade 3 and D. Chromosome 1 in clade 4 compared with all chromosomes in the remaining clades.

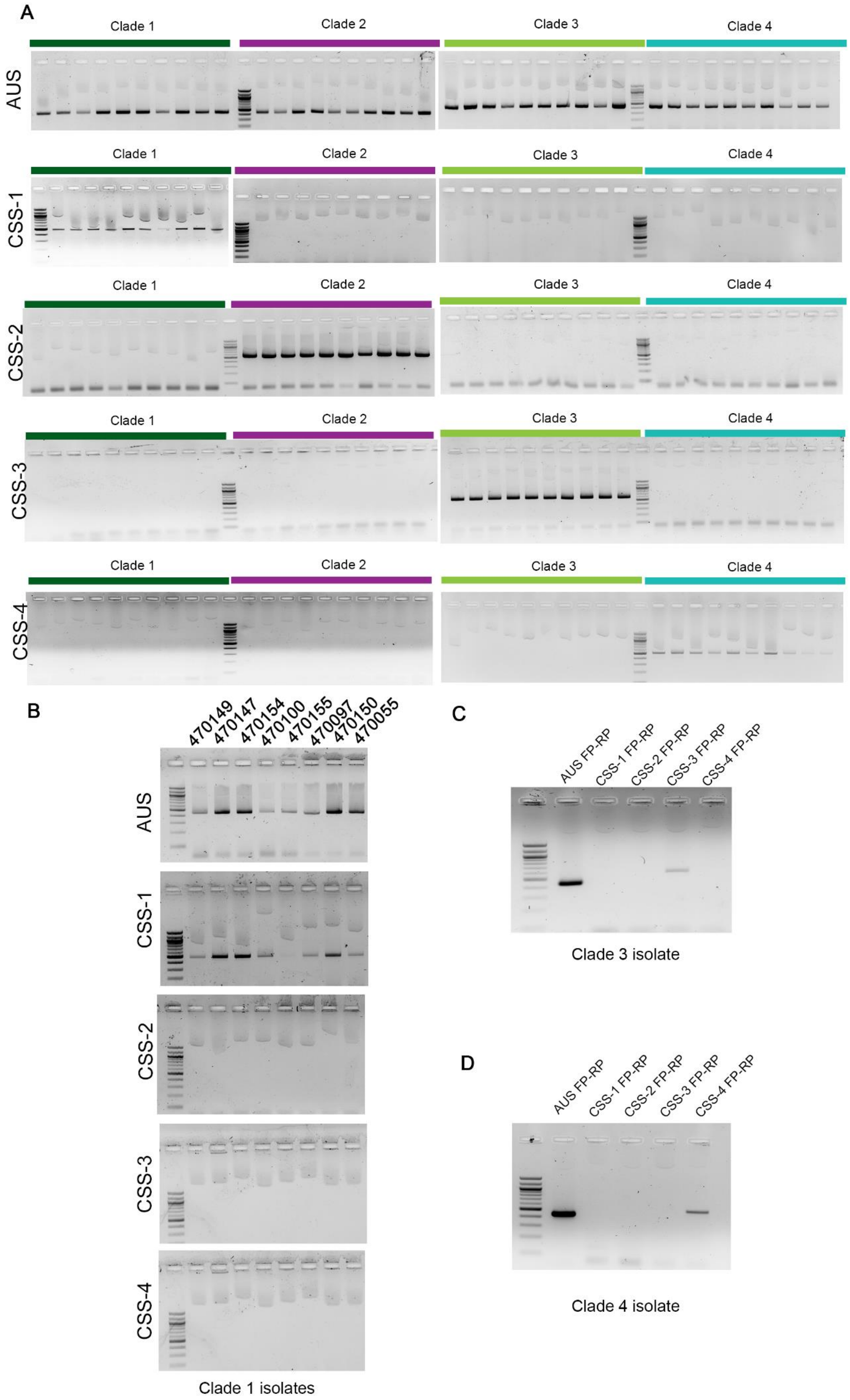
A
Clade 1
Clade 2
Clade 3
Clade 4
AUS
CSS-1
CSS-2
CSS-3
CSS-4
B
470149
470147
470154
470100
470155
470097
470150
470055
Clade 1 isolates
C
AUS FP-RP
CSS-1 FP-RP
CSS-2 FP-RP
CSS-3 FP-RP
CSS-4 FP-RP
Clade 3 isolate
D
Clade 4 isolate

FIG S2 A. Multiple reactions confirm the reproducibility and specificity of clade-specific amplifications. Ten colonies of the strains belonging to each clade were subjected to colony PCR using the designed primers for AUS, CSS-1, CSS-2, CSS-3, and CSS-4. B. Clinical isolates a.470149 b. 470147 c. 470154 d. 470100 e. 470155 f. 470097 g. 470150 h. 470055 were tested with the primer pairs for AUS, CSS-1, CSS-2, CSS-3, and CSS-4. C. Clinical isolate 598A was tested with the primer pairs for AUS, CSS-1, CSS-2, CSS-3, and CSS-4. D. LMDM1219 was tested with the primer pairs for AUS, CSS-1, CSS-2, CSS-3, and CSS-4.

Supplementary Information

CSS-1 (length = 500 bp)

TTATTTGGTCTTCAATCATTGATTCCTTGCTTGAAGATTGGCTTGCCGGTATTCCTT
TCCAAGGTTATCTATGATCTCCCTCATCGTTGAATCACCGGATTCCAGGAAAGGTT
GTATAGGCAGCTGTGTGGTAAGCGAGACAGTCAGCTGGGCTTCCTCCGGGGGCC
TATGCATATTTCGCTTTGTGCTCTAAAACCACTGAAATCAAAAACCTAACGTAAT
TTTGTATGAAAATGTCATATTATTGACTTTACACGCTTTTGTTTTTTAAAACAATG
ACAAGAATTTATTTTTTAGGGTACATAATTTGACAGAAATCGGGCGTGTGGTCTA
GTGGTATGATTCTCGCTTTGGGCGACGATCTGGTACACCAGATTGACATACAAAC
ATGCGAGAGGCCCTGGGTTCAATTCCCAGCTCGCCCCTTCTTTTTCTTCACTTCAT
TACTTTCAATTTGAAATCAAGTCTAATCCTTCCTCAATTCCTACTCACTACACGTA

Homology search of CSS-1 as query against all the clades

(a)

Query	Subject: Clade 1	Percent identity	Alignment length	Mismatch	Gap	Query start	Query end	Subject start	Subject end	E-value	Bit score
CSS-1	CP060339.1	100.000	500	0	0	1	500	905001	905500	0.0	924
	CP060339.1	100.000	183	0	0	1	183	1632714	1632532	5.08e-93	339
	CP060339.1	100.000	183	0	0	1	183	2751091	2751273	5.08e-93	339
	CP060339.1	100.000	183	0	0	1	183	3274148	3274330	5.08e-93	339
	CP060339.1	98.113	106	2	0	320	425	3692990	3692885	7.00e-47	185
	CP060344.1	100.000	183	0	0	1	183	387873	388055	5.08e-93	339
	CP060344.1	93.277	119	5	3	318	435	411519	411635	5.45e-43	172
	CP060341.1	100.000	183	0	0	1	183	514423	514605	5.08e-93	339
	CP060341.1	86.842	114	12	2	320	433	1662735	1662845	1.55e-28	124
	CP060343.1	96.850	127	3	1	311	437	337259	337134	1.16e-54	211

(b)

Query	Subject: Clade 2	Percent identity	Alignment length	Mismatch	Gap	Query start	Query end	Subject start	Subject end	E-value	Bit score
CSS-1	CP043532.1	99.373	319	2	0	182	500	1685470	1685152	2.71e-165	579
	CP043533.1	100.000	183	0	0	1	183	66424	66242	5.01e-93	339
	CP043533.1	97.826	184	0	4	1	183	1544142	1544322	8.45e-86	315
	CP043533.1	86.842	114	12	2	320	433	10603	10493	1.53e-28	124
	CP043531.1	97.268	183	0	4	1	183	1908763	1908586	5.09e-83	305
	CP043531.1	98.113	106	2	0	320	425	476743	476848	6.91e-47	185
	CP043535.1	96.063	127	3	2	311	437	616603	616727	5.31e-53	206
	CP043537.1	93.277	119	5	3	318	435	368603	368719	5.38e-43	172

(c)

Query	Subject: Clade 3	Percent identity	Alignment length	Mismatch	Gap	Query start	Query end	Subject start	Subject end	E-value	Bit score
CSS-1	CP060370.1	90.179	336	16	5	182	500	895701	896036	4.86e-118	422
	CP060369.1	100.000	183	0	0	1	183	14993	15175	5.03e-93	339
	CP060369.1	86.842	114	12	2	320	433	23656	23546	1.53e-28	124
	CP060372.1	96.850	127	3	1	311	437	331151	331026	1.15e-54	211
	CP060367.1	98.113	106	2	0	320	425	489009	489114	6.94e-47	185
	CP060373.1	92.437	119	6	3	318	435	405007	405123	2.51e-41	167

(d)

Query	Subject: Clade 4	Percent identity	Alignment length	Mismatch	Gap	Query start	Query end	Subject start	Subject end	E-value	Bit score
CSS-1	CP043442.1	87.906	339	21	8	182	500	887976	888314	8.34e-106	381
	CP043442.1	94.536	183	3	5	1	183	2150717	2150542	4.05e-74	276
	CP043447.1	98.361	183	3	0	1	183	589897	589715	5.13e-88	322
	CP043447.1	93.277	119	5	3	318	435	554623	554507	5.46e-43	172
	CP043446.1	96.825	126	3	1	311	436	332657	332533	4.16e-54	209
	CP043444.1	86.842	114	12	2	320	433	121209	121099	1.55e-28	124

CSS-2 (length =551 bp)

AGCTACACAAAATGGTTTTTTCAGATTTTAAGAATAACACAAGTCGAATAAAAGG
GGCTTTGCTTTCTCATCACCAACCCCCTCGTTGTCTTGTCACAAAATTCTCATTTT
GGATAATTATTCAAAGATTGTTAGAGTGCGCCGCTTTCTACAAACGGCTGGAAG
ATAGAGCCGCTTGGAAGGAAGGCACCATGCAACCTGTCGCGGATGCCTCTAGTG
GGCACTCCACTTGCGCTTCCACCAATCAAGTGGAGATAAGCTATCGACATCAAGG
TCATAAAGAGGGCGAGGAACAATACAATTATACTACACTTCTTGTTTGTGGCAAC
GAAAATATGCAAATGGATAGTGCAGCATGACACATGCACAAACGATGGGCTATC
ATAAACGCGTAATAAAGATTTCCTTGCATCTTTTCATCAGTTCCGATGAAATATAA
ATTGACTCATCGTCCTCATGCTCTTGATCTCTCCATCCCCTGATATCAGTACATCAT
GACATATATGCGATGTCCCCAGCCTTCAAACTCTACTACTTTG GCATATGATGTG

Homology search of CSS-2 as query against all the clades

Query	Subject: Clade 3	Percent identity	Alignment length	Mismatch	Gap	Query start	Query end	Subject start	Subject end	E-value	Bit score
CSS-2	CP043534.1	100.00	551	0	0	1	551	14050	14600	0.0	1018

CSS-3 (length = 598 bp)

CGATGAGAAACCCCCATCCAAGGATATATTGTTGGTTTCTTTTTTATTATGTTCGTG
CAGTTGAGTGTAAACAATTGTGAACTCAGTGTAGATCCCACATTGGCTGCGAAAAT
GTACTATGGTTCATACCCAGTAATGGAATTATACCACCACCTCTTTTCGCTAGGAAA
CAATATGAATGAGGCCTTTGTGAAAAATGGGATGAAAAAAAAGAAAGGCTAGAGA
AGGATACAAAGCTCAAATTCTGTTTTGGAGCCTACATCAGCGAATGAAATTGGCTG
GTTGATACATGTCGCTCACGGTCTTCTGTTGGCTGCGAAAAAATGACAGAACTTAG
TTTCTTGATAGCAAGTTTACTTTACCTGGCCACAACTTGAACCAGAGTATTGCATC
AAATCTGAATTCACAGAAGTTCTCGAGCCCTCAAAGGTCCGTTTCCCCACAAATGA
GCCAGTTTTTATATACATTGAAAATTGAACAGTCGCAGCCATCTCGCAGCCATCAT
TAGTCCTCTGCAGCTTCCATACACACAAGTTGAAGAAACATGACTGAGTCAAGTCC
ACGATGGTCGTATTGTATTGACTGATAGAAATGAAAA

Homology search of CSS-3 as query against all the clades

Query	Subject	Percent identity	Alignment length	Mismatch	Gap	Query start	Query end	Subject start	Subject end	E-value	Bit score
CSS-3	Clade 1 CP060339.1	95.522	402	10	6	205	598	290350	290751	0.0	636
	Clade 2 CP043532.1	97.574	371	9	0	228	598	2291889	2291519	0.0	636
	Clade 3 CP060370.1	100.000	598	0	0	1	598	287703	288300	0.0	1105
	Clade 4										

	CP043442.1	95.037	403	10	4	205	598	282704	283105	4.21e-179	625

CSS-4 (length = 502 bp)

GGGGGTTTTACTATATAAATTTGTATAGCTTTAAAGCTGAGGAAGAGATGTTAACA
TCTTGACCTTAGCGACCCCAATCAATGTATCCAAAAAAAAAGCCGGCAACACTG
AAATCCTAACGCCTCTAATACATGAAGTAAAAAAAAAAAAGCCGGTTTGGGGGGA
AAAAAGTAGCTATGATTTTAGCCCAATAAAAGTGACGATGGTGATTGAAGGTGG
ATCAAAACTAAAGTACCGGATTAGATTAGTCAGGGTCAAGCTTGAGCTTTCTAAT
TAGTCTAATGCACAAAAGTTGAGCCACTCAAAAGGCGCTGCGCAAGATTAGTCC
ATGTGGGCTGTAATTTAGTGATAATTTAGTGATACAGTTTGTTCAATCGTCTTGGA
TGGAAAGGTTGACAATGCGTCGTGCTAGCTGCTGTATTCTTTTCTTTTTCCGCAA
CATTCTGAATGTATCGTCACTAGTAGCTGAAAGGAGGTTGGGATGAAAATCCCG
ACCTACATAG

Homology search of CSS-4 as query against all the clades

(a)

Query	Subject: Clade 1	Percent identity	Alignment length	Mismatch	Gap	Query start	Query end	Subject start	Subject end	E-value	Bit score
CSS-4	CP060339.1	95.041	363	9	4	149	502	3693128	3693490	2.78e-160	562

(b)

Query	Subject: Clade2	Percent identity	Alignment length	Mismatch	Gap	Query start	Query end	Subject start	Subject end	E-value	Bit score
CSS-4	CP043531.1	95.868	363	6	4	149	502	476605	476243	2.73e-165	579
	CP043531.1	91.000	100	6	3	1	97	1232011	1231912	9.17e-31	132
	CP043532.1	95.652	92	0	2	1	88	1352053	1352144	1.18e-34	145
	CP043534.1	91.011	89	1	3	16	97	71172	71260	3.32e-25	113

(c)

Query	Subject: Clade 3	Percent identity	Alignment length	Mismatch	Gap	Query start	Query end	Subject start	Subject end	E-value	Bit score
CSS-4	CP060367.1	95.592	363	7	4	149	502	488871	488509	1.27e-163	573

(d)

Query	Subject: Clade 4	Percent identity	Alignment length	Mismatch	Gap	Query start	Query end	Subject start	Subject end	E-value	Bit score
CSS-4	CP043442.1	100.000	502	0	0	1	502	3696899	3697400	0.0	928
	CP043442.1	94.175	103	0	3	1	97	2237934	2237832	7.15e-37	152
	CP043442.1	94.175	103	0	3	1	97	2481723	2481621	7.15e-37	152
	CP043442.1	97.753	89	0	2	2	88	3286656	3286744	7.15e-37	152
	CP043442.1	93.333	105	3	3	1	101	3609109	3609213	7.15e-37	152
	CP043442.1	94.175	103	0	3	1	97	3696341	3696443	7.15e-37	152
	CP043442.1	94.118	102	0	4	1	97	1915087	1915187	2.57e-36	150
	CP043442.1	93.269	104	0	3	1	97	1178875	1178978	3.32e-35	147
	CP043442.1	93.204	103	1	3	1	97	2177087	2176985	3.32e-35	147
	CP043442.1	93.269	104	0	3	1	97	2220044	2220147	3.32e-35	147
	CP043442.1	93.204	103	1	3	2	98	2280383	2280485	3.32e-35	147
	CP043442.1	93.269	104	0	3	1	97	2960947	2961050	3.32e-35	147
	CP043442.1	93.269	104	0	3	1	97	3437676	3437779	3.32e-35	147
	CP043442.1	92.453	106	0	5	2	101	3287064	3287167	1.20e-34	145
	CP043442.1	92.233	103	0	4	2	97	3609221	3609322	5.56e-33	139
	CP043442.1	92.079	101	2	4	2	97	2924966	2925065	2.00e-32	137
	CP043446.1	95.098	102	0	3	1	97	563590	563691	5.52e-38	156
	CP043444.1	95.050	101	1	2	2	98	1303960	1303860	5.52e-38	156
	CP043444.1	93.137	102	0	4	2	97	919070	919170	4.30e-34	143
	CP043444.1	92.233	103	0	4	2	97	1303257	1303156	5.56e-33	139
	CP043445.1	95.050	101	0	3	2	97	579469	579569	1.99e-37	154
	CP043445.1	92.233	103	0	4	2	97	969324	969425	5.56e-33	139
	CP043443.1	94.175	103	0	3	1	97	643961	644063	7.15e-37	152
	CP043443.1	93.269	104	0	3	1	97	794351	794454	3.32e-35	147
	CP043443.1	93.269	104	0	3	1	97	1020268	1020165	3.32e-35	147

	CP043443.1	93.269	104	0	3	1	97	1408089	1408192	3.32e-35	147
	CP043443.1	93.137	102	0	4	2	97	1408448	1408548	4.30e-34	143
	CP043448.1	93.269	104	0	3	1	97	320616	320513	3.32e-35	147
	CP043448.1	93.269	104	0	3	1	97	391234	391131	3.32e-35	147
	CP043447.1	93.269	104	0	3	1	97	576411	576514	3.32e-35	147
	CP043447.1	93.269	104	0	3	1	97	586691	586794	3.32e-35	147
	CP043447.1	93.204	103	1	3	1	97	651460	651358	3.32e-35	147
